# Plasma proteomic signatures in HIV-infected individuals post-SARS-CoV-2 infection

**DOI:** 10.1186/s12879-025-12307-1

**Published:** 2025-12-09

**Authors:** Chayanin Chanthara, Janya Khattiya, Sittiruk Roytrakul, Chareeporn Akekawatchai, Narumon Phaonakrop, Nattamon Niyomdecha

**Affiliations:** 1 Graduate Program in Medical Technology, Faculty of Allied Health Sciences, Thammasat University, Rangsit Campus, Pathum Thani, Bangkok, Thailand; 2 Department of Disease Control, Ministry of Public Health, Office of Disease Prevention and Control Region 11, Nakhon Si Thammarat, Thailand; 3 Thammasat University Research Unit in Diagnostic Molecular Biology of Chronic Diseases Related to Cancer (DMB-CDC), Pathum Thani, Thailand; 4 National Center for Genetic Engineering and Biotechnology, National Science and Technology Development Agency, Pathum Thani, Thailand; 5 Department of Medical Technology, Faculty of Allied Health Sciences, Thammasat University, Rangsit Campus, Pathum Thani, Bangkok, Thailand

**Keywords:** Plasma proteomics, HIV, SARS-CoV-2, Biomarkers, Immune dysregulation

## Abstract

The long-term immunological effects of prior Severe Acute Respiratory Syndrome Coronavirus 2 (SARS-CoV-2) infection in people living with Human Immunodeficiency Virus (HIV) remain poorly understood. This study aimed to characterize plasma proteomic alterations associated with previous SARS-CoV-2 infection in HIV-infected individuals and to identify potential biomarkers and affected pathways. High-throughput liquid chromatography–tandem mass spectrometry (LC–MS/MS) was performed on plasma obtained from three groups: HIV-infected individuals with documented prior SARS-CoV-2 infection, HIV-monoinfected individuals, and healthy controls. A total of 13,675 proteins were identified. Hierarchical clustering and sparse partial least squares discriminant analysis revealed distinct proteomic profiles in the prior-SARS-CoV-2 group. Ten proteins with the highest discriminatory power—PRR11, TEX14, METTL9, NMD3, PXT1, CRISP2, MELK, SPF27, GCP6, and GTPBP8—were associated with cell cycle regulation, RNA processing, apoptosis, mitochondrial function, and cytoskeletal organization. Subcellular localization indicated predominant nuclear and cytoplasmic involvement, suggesting alterations in transcriptional regulation and intracellular structural dynamics. These signatures imply that preceding SARS-CoV-2 exposure may compound HIV-associated immune dysregulation and disrupt cellular homeostasis. The findings offer novel molecular insights into the persistent biological impact of SARS-CoV-2 in the context of HIV and identify candidate proteomic biomarkers with potential utility for risk stratification and targeted intervention in immunocompromised populations.

**Supplementary Information:**

The online version contains supplementary material available at 10.1186/s12879-025-12307-1.

## Introduction

Severe Acute Respiratory Syndrome Coronavirus 2 (SARS-CoV-2) is responsible for Coronavirus Disease 2019 (COVID-19), a significant contributor to morbidity and mortality, particularly among individuals living with HIV (PLWH). The likelihood of developing COVID-19 among PLWH is approximately 38% higher than in those without HIV infection [1]. Among hospitalized COVID-19 patients, studies demonstrated that PLWH aged under 65, particularly those with a CD4 + cell count less than 350 cells/mm³, experience increased in-hospital mortality risk compared to age-similar general population cohorts. A direct dose-response relationship underscores this vulnerability, with mortality risk increasing markedly at CD4 + counts under 200 cells/mm³ [2–4].

The immunocompromised state and chronic inflammation in HIV patients are characterized by CD4 + T cell depletion, CD8 + T cell expansion, and persistent immune activation [5]. SARS-CoV-2 can efficiently infect CD4 + T cells by binding to cell entry receptors other than angiotensin-converting enzyme 2 (ACE2), subsequently inducing apoptosis [6]. SARS-CoV-2 is often associated with a cytokine storm or cytokine release syndrome (CRS), which is characterized by the excessive production of pro-inflammatory cytokines, including IL-6, IL-1β, and TNF-α. The pronounced inflammatory response results in extensive systemic inflammation, acute respiratory distress syndrome (ARDS), multi-organ dysfunction, and is a significant predictor of elevated mortality rates. This exaggerated immune response converts a protective host mechanism into a pathogenic process [7]. A meta-analysis indicated that HIV infection correlates with a heightened risk of developing COVID-19 cytokine release syndrome (Relative Risk [RR] = 1.48). The increased incidence of CRS in PLWH is associated with a higher probability of admission to intensive care units and the requirement for mechanical ventilation, both of which are significant indicators of severe disease progression [8]. Consequently, the interaction between HIV and SARS-CoV-2 may align in their impact on the immune system, presenting a considerable challenge to immunopathogenesis.

The relationship between SARS-CoV-2 and HIV infections is not yet fully understood, despite a growing body of evidence regarding COVID-19 in the general population. The alterations in soluble proteomic markers post-SARS-CoV-2 infection provide insights into the immune responses in PLWH who have COVID-19. This study investigates the proteomic expression patterns in plasma samples from HIV-positive patients with a documented prior SARS-CoV-2 infection, compared to those with HIV-monoinfection and healthy controls, utilizing high-throughput proteomics technologies. This study will improve our understanding of the molecular pathways involved in the pathogenesis of SARS-CoV-2 infection within the framework of HIV-related immunosuppression. The findings may aid in identifying novel biomarkers for early diagnosis and prognostication, thereby contributing to the development of precise diagnostic tools and effective therapeutic or vaccine strategies for this vulnerable population.

## Materials and methods

### Participants

**Fig. 1:**
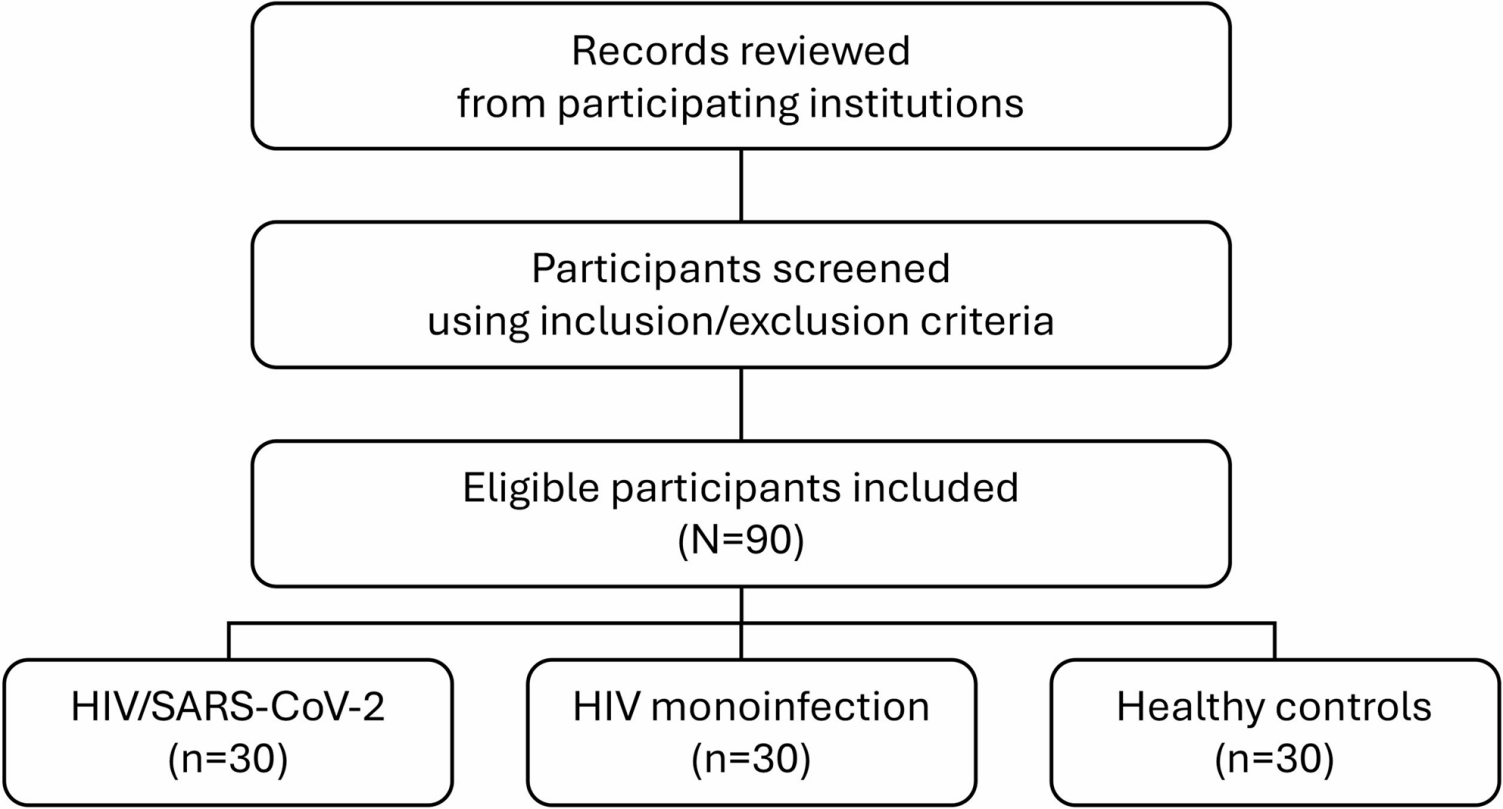
Participant screening and selection flowchart. This flowchart summarizes the screening and selection process for this pilot cross-sectional study. Medical records and residual plasma samples from three participating institutions were reviewed, after which participants were screened according to predefined inclusion and exclusion criteria. A total of 90 eligible participants (30 per group) were included and systematically allocated into three groups: HIV/SARS-CoV-2 (*n* = 30), HIV monoinfection (*n* = 30), and healthy controls (*n* = 30)

This pilot cross-sectional study using retrospective data included a total of 90 participants, systematically allocated into three groups (30 participants per group) based on infection status: individuals with HIV infection and a history of SARS-CoV-2 infection (hereafter referred to as HIV/SARS-CoV-2), individuals with HIV monoinfection, and healthy uninfected controls. An overview of the participant screening and eligibility process is presented in Fig. 1. Given the exploratory nature of this pilot study, a formal sample size calculation was not required. Instead, the target of 30 individuals per group was determined pragmatically based on the availability of eligible residual plasma specimens meeting the inclusion criteria during the study period—a sample size commonly applied in discovery-scale proteomic investigations. The study utilized medical records and residual plasma samples obtained from Nakhon Si Thammarat Municipal Hospital, Maharaj Nakhon Si Thammarat Hospital, and the Regional Disease Control Office No. 11, all located in Nakhon Si Thammarat Province, Thailand. Data and samples were collected between October 1, 2022, and September 30, 2024.

Eligibility criteria for both HIV-infected groups included age ≥ 18 years, receipt of antiretroviral therapy (ART) for at least 6 months, HIV viral load < 20 copies/mL, and CD4 + T lymphocyte count > 200 cells/mL. For the HIV/SARS-CoV-2 group, an additional requirement was a documented history of laboratory-confirmed SARS-CoV-2 infection with complete clinical recovery. Exclusion criteria for both groups included co-infection with hepatitis B or C viruses, presence of opportunistic infections, insufficient plasma volume or poor sample quality, incomplete clinical data, and any history of malignancy, cardiovascular disease, diabetes mellitus, or autoimmune disorders—conditions known to influence systemic inflammatory responses. In cases of uncertainty, participants were excluded and noted for consideration in the study’s limitations.

Healthy controls were required to be ≥ 18 years of age with no prior history of HIV or SARS-CoV-2 infection and negative screening for hepatitis B and C viruses. The same exclusion criteria applied. Plasma samples were systematically selected based on these predefined criteria and assessed for volume sufficiency and sample integrity prior to inclusion in the proteomic analysis.

Baseline demographic and clinical data were extracted from medical records maintained by the Medical Technology Departments of the participating institutions. Collected information included demographic characteristics (sex, age), antiretroviral therapy regimen and treatment duration, and laboratory parameters at the time of diagnosis, such as hepatitis B and C infection status, HIV viral load, CD4 + T lymphocyte count, and SARS-CoV-2 test results.

### Protein digestion and LC–MS/MS-based proteomic analysis

For proteomic analysis, plasma samples from individuals in each group (HIV/SARS-CoV-2, HIV monoinfection, and healthy controls) were pooled to reduce the effect of biological variation and generate one representative sample per group. A volume of 50 µL per participant was combined to produce a single pooled sample for downstream LC–MS/MS analysis. This pooling strategy is commonly used in discovery-scale proteomics to increase analytical reproducibility, minimize inter-individual noise, and enable deep proteome coverage under limited specimen availability. Individual-level mass spectrometry analysis could not be performed because the residual clinical plasma specimens were available only in small aliquots that were insufficient for both comprehensive proteomic profiling and confirmatory assays. All procedures were performed in accordance with institutional biosafety guidelines and approved by the Institutional Biosafety Committee (TU-IBC), Thammasat University (Certificate No. 001/2567).

The Lowry assay was employed to quantify the total protein concentration in plasma samples, with bovine serum albumin functioning as the calibration standard. Protein samples were subjected to in-solution enzymatic digestion following quantification. The proteins were initially solubilized in 10 mM ammonium bicarbonate (AMBIC). Subsequently, they were reduced of disulfide bonds using 5 mM dithiothreitol in 10 mM AMBIC at 60 °C for 1 h. The sulfhydryl groups were subsequently alkylated in the presence of 15 mM iodoacetamide in 10 mM AMBIC at room temperature for 45 min in the dark. Proteolytic digestion was performed overnight at 37 °C with sequencing-grade porcine trypsin (Promega Corporation) at a 1:20 enzyme-to-substrate ratio. To streamline the workflow description while maintaining essential methodology, standard steps such as peptide drying and reconstitution were summarized: the resulting tryptic peptides were cleaned, concentrated, and reconstituted in 0.1% formic acid before LC–MS/MS analysis [9]. Peptide analysis was performed using a ZenoTOF 7600 mass spectrometer (SCIEX, Framingham, MA, USA) in conjunction with an Ultimate 3000 Nano/Capillary LC system (Thermo Fisher Scientific, Inc.). Each pooled sample was analyzed in technical triplicate to ensure analytical reproducibility and robustness.

### Bioinformatics and data analysis

Protein quantification for individual plasma samples was conducted using MaxQuant version 2.5.0.0 (Max Planck Institute of Biochemistry), employing the integrated Andromeda search engine to correlate MS/MS spectra with the *Homo sapiens* reference proteome retrieved from the UniProt database [10]. Label-free quantification (LFQ) was performed under MaxQuant’s default parameters, including a maximum of two missed cleavages, a mass tolerance of 0.6 Da for the main search, trypsin as the proteolytic enzyme, carbamidomethylation of cysteine as a fixed modification, and oxidation of methionine along with N-terminal acetylation as variable modifications. Protein identification required peptides to be at least seven amino acids in length, with a minimum of one unique peptide. Proteins were considered valid for further analysis only if they were identified by at least two peptides, including one unique peptide. The protein false discovery rate (FDR) was controlled at 1% using a reversed decoy database, and a maximum of five modifications per peptide was allowed. The *Homo sapiens* reference proteome, downloaded from UniProt on January 6, 2025, was used as the search FASTA file. The raw MS/MS spectral data are available on the ProteomeXchange repository under registration numbers JPST004046 and PXD067809 (https://repository.jpostdb.org/preview/57459627568af83c83697a; Access key: 6382).

Protein intensity values were log₂-transformed and quantile-normalized prior to statistical testing to reduce heteroscedasticity and ensure comparability across proteins. Multiple testing correction for univariate analyses was performed using the Benjamini–Hochberg false discovery rate (FDR) method with a significance threshold of FDR < 0.05. Because analyses were conducted on pooled samples, we acknowledge that FDR-adjusted results reflect group-level rather than individual-level variance.

Functional classification and biological role analysis of differentially expressed proteins were conducted using the PANTHER classification system version 19.0 [11]. To explore group-specific proteomic patterns and identify key discriminatory proteins, statistical analyses were performed using MetaboAnalyst version 6.0 (Wishart Research Group, University of Alberta), a comprehensive web-based platform for mass spectrometry data interpretation. Sparse Partial Least Squares Discriminant Analysis (sPLS-DA) was applied to visualize clustering trends and identify proteins that contributed most to group separation. The optimal number of latent components was determined by five-fold cross-validation based on the lowest balanced classification error rate. To minimize overfitting, the number of selected variables per component was constrained, and model robustness was evaluated using repeated subsampling stability analysis. The loading plot further illustrated the relative contribution of each variable to the principal components. Variable Importance in Projection (VIP) scores were used to rank the proteins according to their influence on model performance, helping prioritize proteins of interest. Additionally, non-parametric analysis of variance was performed using the Kruskal–Wallis test. Proteins showing statistically significant differences among groups were identified based on a p-value < 0.05 and an FDR < 0.05. Identified proteins were further validated and annotated based on the UniProt and PANTHER v19.0 databases, with classification according to molecular function and subcellular localization.

Protein–protein interaction networks were constructed using STITCH version 5.0, which enabled the visualization and prediction of interactions between selected proteins and known interactors within the database, thereby facilitating the interpretation of potential biological pathways and mechanisms underlying observed proteomic differences.

## Results

### Demographic and clinical characteristics of study participants

In Table 1 and Supplementary file S1, a total of 90 participants were enrolled and equally stratified into three groups: individuals with HIV and prior SARS-CoV-2 infection (HIV/SARS-CoV-2) (*n* = 30), individuals with HIV monoinfection (*n* = 30), and healthy controls (*n* = 30). The median age in the healthy control group was significantly lower than that of the HIV/SARS-CoV-2 and HIV monoinfection groups. A statistically significant difference in gender distribution was noted among the groups (*p* = 0.036). The proportion of female participants was highest in the HIV/SARS-CoV-2 group (66.7%), followed by the HIV monoinfection (50.0%) and healthy control (33.3%) groups. Conversely, the proportion of male participants increased from 33.3% in the HIV/SARS-CoV-2 group to 66.7% in the healthy controls.

**Table 1:** Demographic and clinical characteristics of study participants stratified by group (*n* = 30 per group)

Characteristics	Frequency, *n* (%) or Median (range)			*P*-VALUE
	HIV/SARS-CoV-2 (*n* = 30)	HIV Monoinfection (*n* = 30)	Healthy Control (*n* = 30)	
Age (yeas)	45.50 (21–75)	48.50 (25–62)	28 (17–51)	**< 0.001**
Gender				**0.036**
Male	10 (33.33%)	15 (50.00%)	20 (66.67%)	
Female	20 (66.67%)	15 (50.00%)	10 (33.33%)	
Duration from infection onset to sampling HIV (Year) SARS-CoV-2 (Month)	12 (1–21) 22 (1–35)	9.5 (1–20) -	- -	**0.773** **N/A**
Undetectable HIV Viral load (< 20 copies/mL)	30 (100%)	30 (100%)	-	**N/A**
Absolute CD4 count (cells/mm³)	658.00 (133-2,137)	818.50 (284-1,608)	-	**0.036**
Duration of ARV Treatment (Month)	125.50 (6-242)	120.00 (10–215)	-	**0.790**
ARV drug ^a^				
Tenofovir/Lamivudine or Zidovudine/ Dolutegravir	30 (100%)	30 (100%)	-	**N/A**

Significant level at P-value < 0.05

^a^ Tenofovir/Zidovudine/Dolutegravir is designed for use during pregnancy

Abbreviations: ARV, Antiretroviral drug; CD4, CD4^+^ T-lymphocyte; -, No data; and N/A, Not Applicable

N/A: Statistical comparison not applicable due to absence of variability or data available in only one group

The duration of antiretroviral therapy (ART) was comparable between the HIV/SARS-CoV-2 and HIV monoinfected groups, with median durations of 125.5 months (range: 6–242) and 120.0 months (10–215), respectively (*p* = 0.790). All HIV-positive participants were administered a standardized ART regimen of Tenofovir, Lamivudine, and Dolutegravir, with the exception of pregnant women who received Zidovudine in place of Lamivudine. The assessment of viral load and immunological status on the sampling day indicated that all samples exhibited undetectable HIV levels (< 20 copies/mL). The absolute CD4 + T-cell count was significantly lower in the HIV/SARS-CoV-2 group (median: 658 cells/mm³; range: 133–2,137) compared to the HIV monoinfection group (median: 818.5 cells/mm³; range: 284–1,608), suggesting a potential additive effect of SARS-CoV-2 infection on immune suppression (*p* = 0.036).

### Differential proteomic profiles across HIV, HIV/SARS-CoV-2, and control groups

**Fig. 2:**
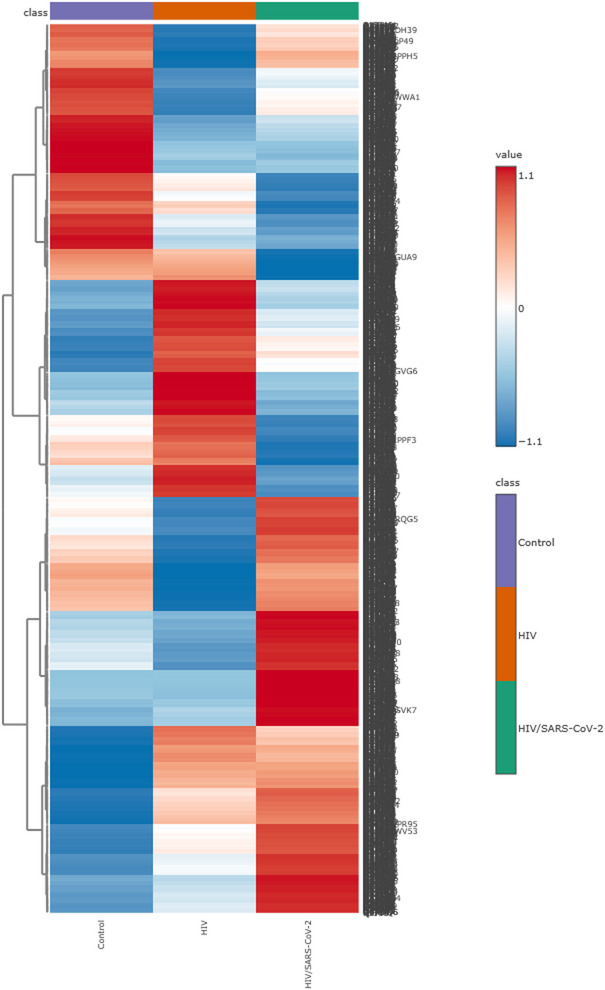
Hierarchical clustering heatmap of protein expression profiles across study groups. This heatmap depicts standardized expression levels (z-scores) for proteins identified from pooled plasma samples (one pooled sample per group, derived from 30 individuals per group). Rows represent individual proteins, and columns represent the three pooled samples corresponding to each study group. Hierarchical clustering was applied to visualize group-specific expression patterns. Red indicates upregulated expression, blue indicates downregulation, and white indicates average expression

A total of 13,675 proteins across HIV, HIV/SARS-CoV-2, and control groups were identified by LC-MS/MS. Protein expression analysis revealed distinct proteomic profiles across the three study groups. As shown in Fig. 2, hierarchical clustering of z-score normalized protein expression data revealed discrete clustering of participants according to infection status. The HIV/SARS-CoV-2 group exhibited pronounced upregulation of several proteins compared to both the HIV-monoinfected and healthy control groups. In contrast, the control group demonstrated consistent downregulation of the same protein clusters. These proteomic expression patterns suggest enhanced immune activation and potential inflammatory dysregulation in HIV/SARS-CoV-2 individuals, highlighting the synergistic impact of HIV and SARS-CoV-2 infection on host gene expression.

**Fig. 3:**
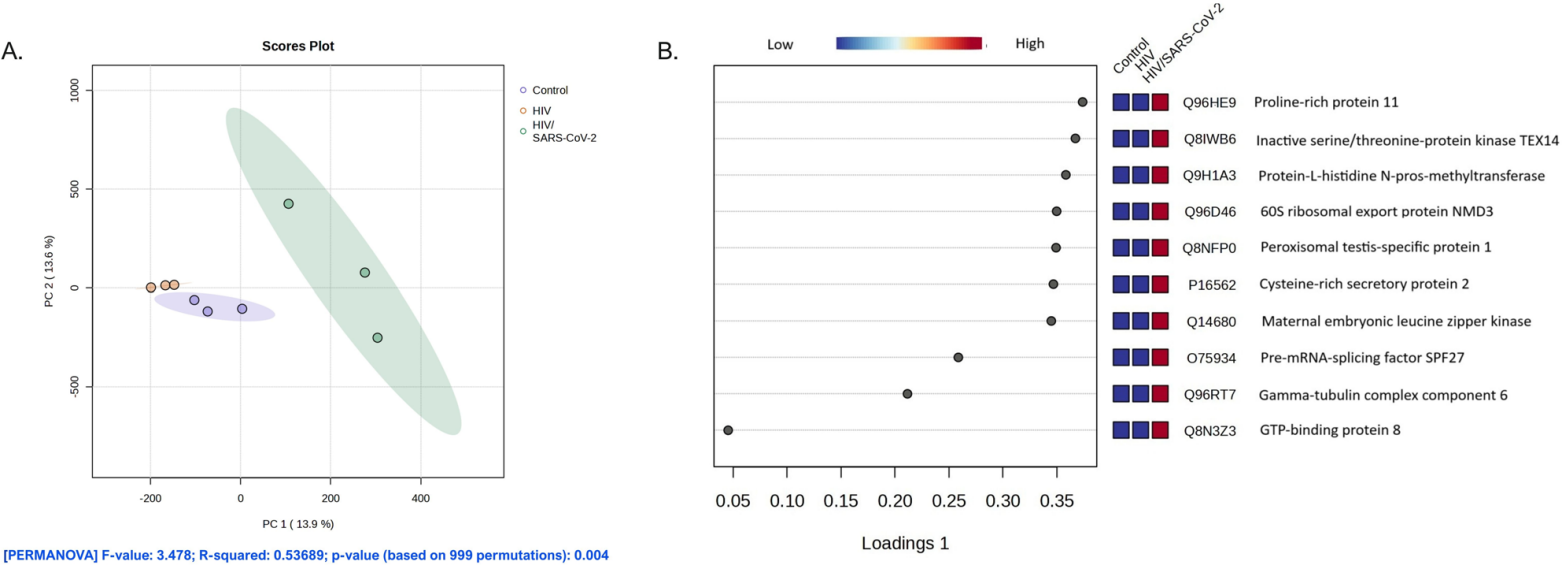
The multivariate analysis of sPLS-DA demonstrates diverse molecular signatures among various clinical groups. (**A**) The score plot from sPLS-DA demonstrates distinct separation among the control, HIV, and HIV/SARS-CoV-2 groups. Each group contained one pooled plasma sample derived from 30 individual participants, analyzed in three independent LC–MS/MS technical replicates, generating the three points visualized per group. PERMANOVA analysis indicates a statistically significant group effect (F = 3.478, R² = 0.53689, *p* = 0.004). (**B**) The sPLS-DA loadings figure delineates the ten most discriminatory proteins that facilitate group separation. The highest-ranked features based on absolute loading values are shown, accompanied by heatmap representations illustrating relative expression across the three pooled samples

The differential clustering patterns observed in the heatmap prompted the application of sparse Partial Least Squares Discriminant Analysis (sPLS-DA) to further evaluate the discriminatory capacity of the proteomic features and identify the most influential biomarkers driving group separation. As shown in Fig. 3A, the sPLS-DA scores plot demonstrates clear separation of the HIV/SARS-CoV-2 group from the other two groups along the first two latent components, which account for 13.9% and 13.6% of the total variance, respectively. This multivariate model represents a three-group comparison, as it simultaneously incorporates all study groups within a single classification framework rather than relying on pairwise contrasts. Although moderate overlap was observed between the control and HIV groups, the HIV/SARS-CoV-2 group exhibited a distinctly segregated distribution, indicating unique molecular perturbations associated with prior SARS-CoV-2 infection in the context of HIV. Collectively, these findings support the presence of a distinct proteomic signature in the HIV/SARS-CoV-2 group, underscoring the potential utility of proteomic biomarkers for differentiating disease states and informing targeted clinical or mechanistic investigations.

**Fig. 4:**
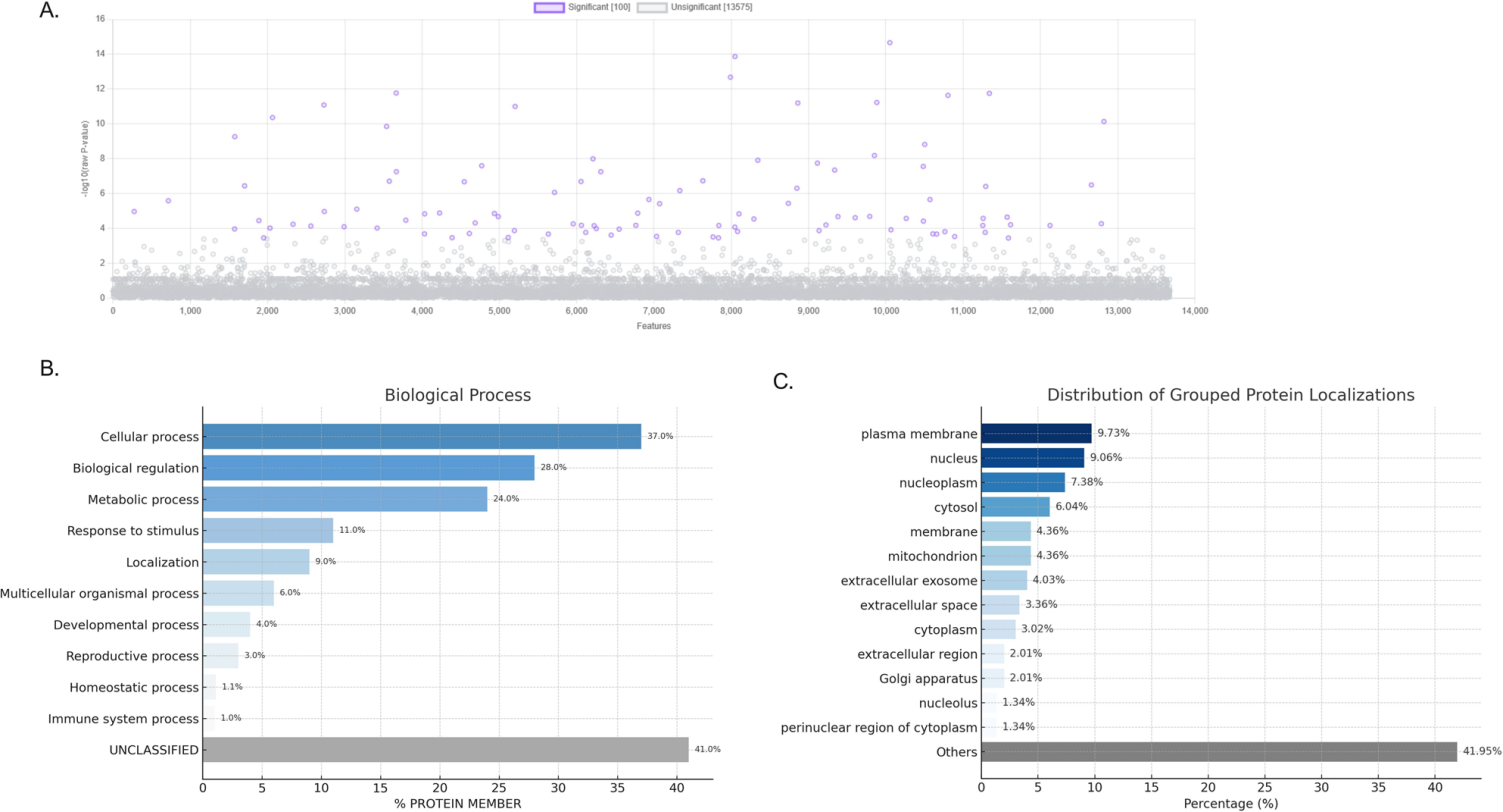
Proteomic identification of discriminative proteins with functional and subcellular localization profiling (based on pooled samples; 30 individuals per group). (**A**) A significance scatter plot displaying –log₁₀(p-values) for 13,675 proteins, with 100 features attaining statistical significance (*p* < 0.05, adjusted) highlighted in purple The x-axis denotes individual protein characteristics, whilst the y-axis signifies –log10-transformed p-values derived from the statistical analysis. (**B**) Functional enrichment analysis of the top 100 important proteins utilizing the PANTHER (Protein ANalysis THrough Evolutionary Relationships) annotation databases. The bars indicate the percentage of proteins linked to each Gene Ontology (GO) term. (**C**) Distribution of grouped protein localizations, highlighting the proportion of proteins assigned to each subcellular compartment. The “Others” category includes proteins with unclassified or miscellaneous localizations. Graphs were created using ChatGPT’s built-in Python visualization feature (Matplotlib-based)

One-way ANOVA, specifically the Tukey’s Post Hoc Test, was utilized to determine statistically significant differences in peak intensities among three experimental groups. Figure 4A, and Supplementary file S2 illustrate the results through a significance scatter plot, where –log₁₀(p-values) reflects the level of statistical significance. One hundred characteristics above the FDR-adjusted significance criterion (*p* < 0.05), indicating their possible role in pathophysiological variations among all groups. These significant characteristics are appropriate for biomarker identification.

A Panther Gene Ontology (GO) analysis was conducted to investigate the biological significance of these notable proteins. Figure 4B illustrates that a considerable percentage of the identified important proteins are predominantly engaged in fundamental biological processes. The predominant GO term was “cellular process,” accounting for 37.0% of all annotated protein members. This was succeeded by “biological regulation” and “metabolic process,” with 28.0% and 24.0%, respectively. In the biological context under investigation, these findings suggest a significant involvement of fundamental cellular machinery. Additional contributions were observed from categories such as “response to stimulus” (11.0%), “localization” (9.0%), and “multicellular organismal process” (6.0%), demonstrating that signaling and compartmentalization also play a role. Terms that were biologically pertinent but were less represented included “developmental process” (4.0%), “reproductive process” (3.0%), “homeostatic process” (1.1%), and “immune system process” (1.0%). Significantly, 41.0% of the proteins were designated as “unclassified,” underscoring the opportunity for identifying novel or inadequately annotated functional pathways.

To elucidate the subcellular distribution of the proteins identified in our dataset, we performed a localization analysis based on GO annotations (Fig. 4C). The distribution revealed that the most prominent localizations were the plasma membrane (9.73%), nucleus (9.06%), nucleoplasm (7.38%), and cytosol (6.04%), indicating that a considerable proportion of the dysregulated proteins are associated with intracellular signaling, transcriptional regulation, and membrane-associated processes. Additional localizations included the mitochondrion and extracellular compartments, suggesting involvement in metabolic responses and intercellular communication. A significant portion of proteins (41.95%) were classified under “Others,” representing diverse or less-characterized localizations. This spatial mapping supports the hypothesis that HIV/SARS-CoV-2 sample might alter key cellular processes across multiple subcellular compartments, reflecting widespread proteomic reprogramming.

### Key proteomic signatures associated with HIV/SARS-CoV-2 identified by sPLS-DA

**Fig. 5:**
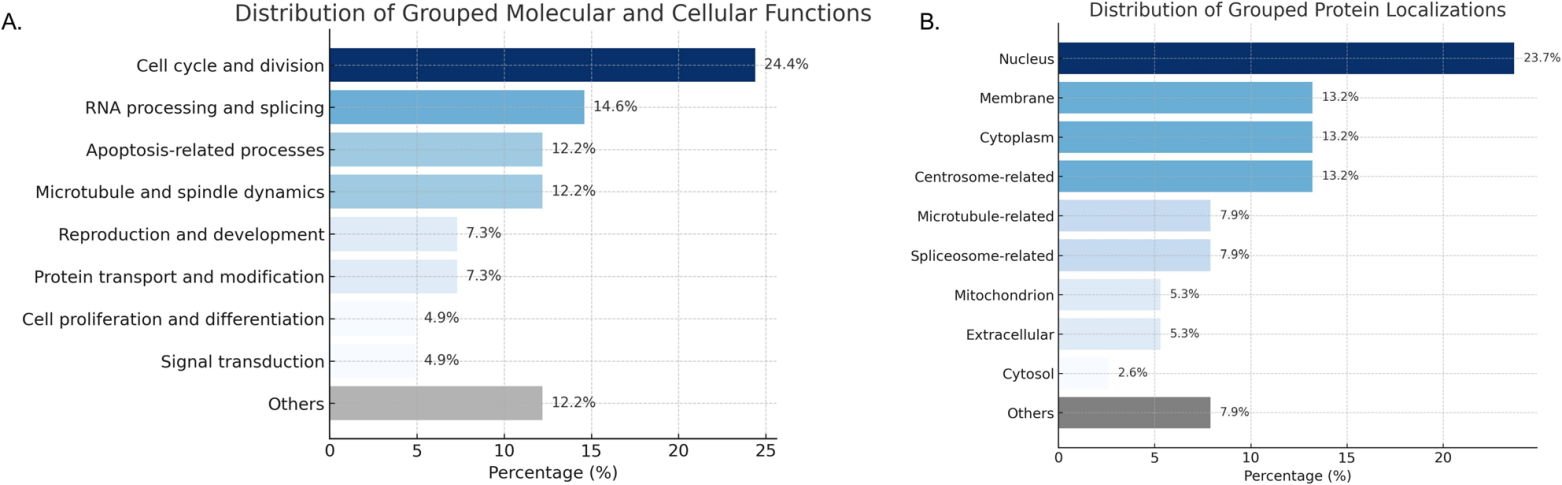
Functional classification of ten significant proteins identified from pooled proteomic profiles (30 individuals per group) based on molecular and cellular roles and subcellular localization. (**A**) Distribution of grouped molecular and cellular functions. (**B**) Subcellular localization of the significantly altered proteins. Bars are colored according to the relative percentage of proteins in each category, with darker shades representing higher proportions. Gray bars indicate the “Others” category. Percentages are shown at the end of each bar. Graphs were created using ChatGPT’s built-in Python visualization feature (Matplotlib-based)

To identify proteins most responsible for discriminating between study groups, a sPLS-DA was conducted, and variable importance in projection (VIP) scores (Supplementary file S3 were calculated. As shown in Fig. 3B; Table 2, heatmap visualization of the expression patterns of these top-ranked proteins revealed distinct group-specific expression profiles. The top 10 features contributing to group separation along component 1 were ranked based on their absolute loading values. Proteins with the highest VIP scores also demonstrated consistent directional expression changes across the HIV/SARS-CoV-2, HIV, and control groups, reinforcing their role as discriminatory features despite the absence of individual-level fold-change calculations inherent to the pooled-sample design. These discriminatory proteins were generally more highly expressed in the HIV/SARS-CoV-2 group, supporting the conclusion that prior SARS-CoV-2 exposure induces unique alterations in the circulating proteome. Analysis for localization and function of these proteins was subsequently performed using UniProt and PANTHER databases (Fig. 5). As illustrated in Fig. 5A, the most prominent molecular and cellular functional category was “cell cycle and division” (24.4%), highlighting a strong association with proliferative activity. This was followed by “RNA processing and splicing” (14.6%) and “apoptosis-related processes” (12.2%), suggesting active regulation of gene expression and cell fate. Additional processes such as “microtubule and spindle dynamics” (12.2%) and “reproduction and development” (7.3%) were also represented, further supporting the involvement of cytoskeletal remodeling and developmental pathways. Less frequent but relevant functions included “protein transport and modification” (7.3%), “cell proliferation and differentiation” (4.9%), and “signal transduction” (4.9%). A small subset (12.2%) was grouped under “others,” indicating potential novel or less-characterized functions.

**Table 2:** List of significantly differentially expressed proteins among the control, HIV-monoinfected, and HIV/SARS-CoV-2 groups based on univariate analysis and supervised multivariate analysis (pooled samples; 30 individuals per group)

ID^a^	Entry Name	Protein Name	sPLSDA		ANOVA	
			Absolute loading values	*P*-VALUE	-log10 (*P*-Value)	FDR
Q96HE9	PRR11_HUMAN	Proline-rich protein 11	0.37381	2.17E-15	14.663	2.97E-11
Q8IWB6	TEX14_HUMAN	Inactive serine/threonine-protein kinase TEX14 (Protein kinase-like protein SgK307) (Sugen kinase 307) (Testis-expressed sequence 14) (Testis-expressed sequence 14 protein)	0.36714	2.11E-13	12.676	9.61E-10
Q9H1A3	METL9_HUMAN	Protein-L-histidine N-pros-methyltransferase (EC 2.1.1.-) (DORA reverse strand protein) (DREV) (DREV1) (Methyltransferase-like protein 9) (hMETTL9)	0.3583	1.78E-12	11.75	4.87E-09
Q96D46	NMD3_HUMAN	60 S ribosomal export protein NMD3 (hNMD3)	0.34975	5.92E-12	11.228	1.08E-08
Q8NFP0	PXT1_HUMAN	Peroxisomal testis-specific protein 1 (Small testis-specific peroxisomal protein)	0.34921	6.29E-12	11.201	1.08E-08
P16562	CRIS2_HUMAN	Cysteine-rich secretory protein 2 (CRISP-2) (Cancer/testis antigen 36) (CT36) (Testis-specific protein TPX-1)	0.34675	8.22E-12	11.085	1.25E-08
Q14680	MELK_HUMAN	Maternal embryonic leucine zipper kinase (hMELK) (EC 2.7.11.1) (Protein kinase Eg3) (pEg3 kinase) (Protein kinase PK38) (hPK38) (Tyrosine-protein kinase MELK) (EC 2.7.10.2)	0.34481	9.99E-12	11	1.37E-08
O75934	SPF27_HUMAN	Pre-mRNA-splicing factor SPF27 (Breast carcinoma-amplified sequence 2) (DNA amplified in mammary carcinoma 1 protein) (Spliceosome-associated protein SPF 27)	0.25865	5.44E-10	9.2642	5.32E-07
Q96RT7	GCP6_HUMAN	Gamma-tubulin complex component 6 (GCP-6)	0.21149	2616.1	8.8231	1.37E-06
Q8N3Z3	GTPB8_HUMAN	GTP-binding protein 8	0.045338	1.22E-08	7.9126	9.29E-06

Proteins with FDR and p-values < 0.05 were considered statistically significant

^a^ UniProt accession code

Abbreviations: FDR, false discovery rate

In parallel, subcellular localization analysis (Fig. 5B) revealed that a substantial proportion of the significant proteins localized to the nucleus (23.7%), consistent with the dominance of nuclear processes such as transcriptional regulation and cell cycle control. Membrane, cytoplasm, and centrosome-related compartments each accounted for 13.2%, reflecting involvement in signaling, trafficking, and microtubule organization. Additional localizations included microtubule- and spliceosome-related components (each 7.9%), mitochondrion and extracellular regions (each 5.3%), and cytosol (2.6%). The “others” category represented 7.9%, encompassing less common or poorly annotated subcellular niches. The centrality of cell division, RNA metabolism, and intracellular structural dynamics in the pathophysiology of the observed protein alterations in the HIV/SARS-CoV-2 sample is underscored by these findings.

### Protein–protein interaction network and functional enrichment analysis reveal dysregulated cellular processes in HIV/SARS-CoV-2

**Fig. 6:**
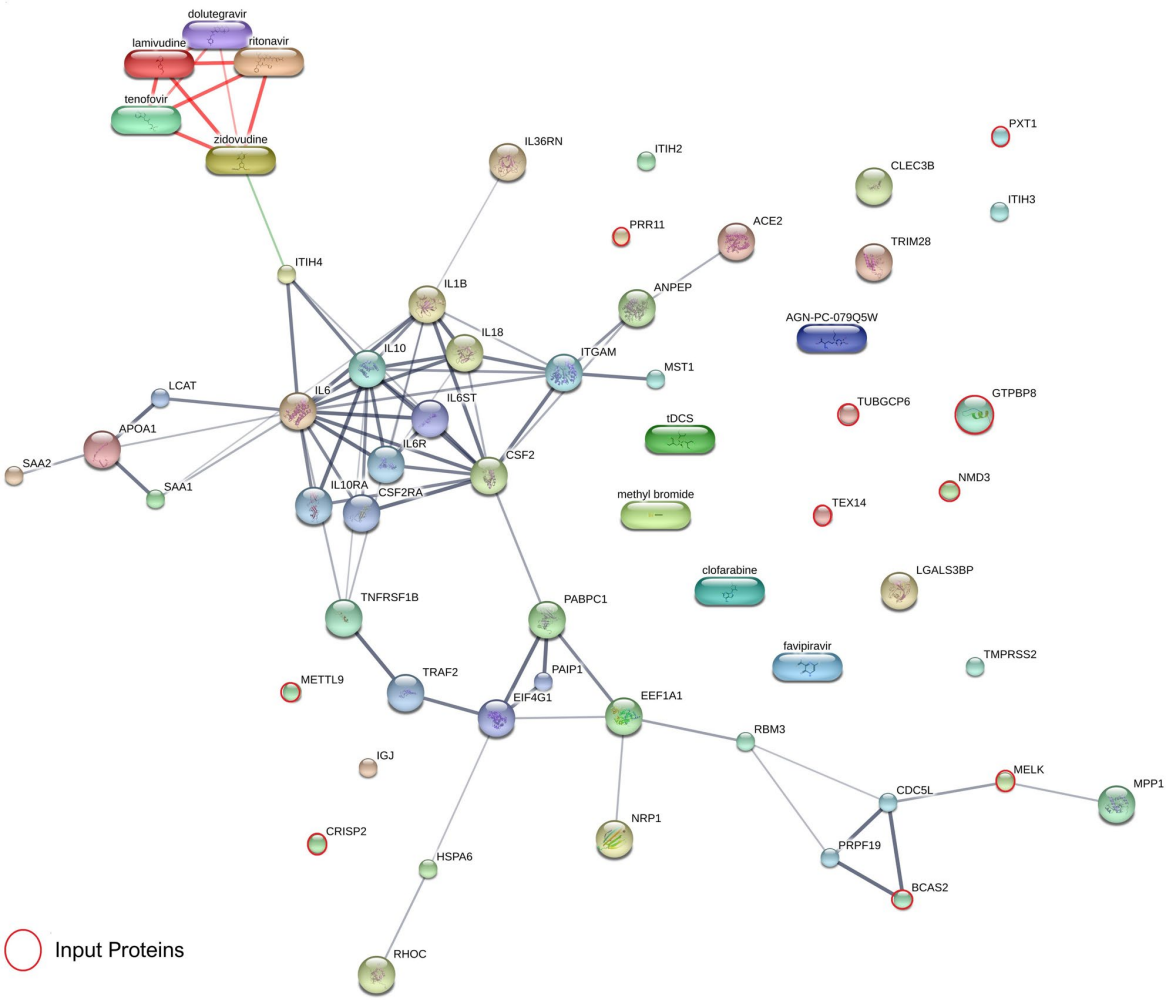
Protein–protein interaction network analysis of significant proteins identified from pooled proteomic datasets and therapeutic agents relevant to HIV/SARS-CoV-2. The network was generated using STITCH (version 5.0; created July 14, 2025) and incorporates significantly altered proteins identified in this study, along with manually curated inputs comprising antiviral drugs and immune mediators. HIV-related drugs (tenofovir, lamivudine, dolutegravir, zidovudine) and SARS-CoV-2 treatments (paxlovid [nirmatrelvir–ritonavir], molnupiravir [Lagevrio], remdesivir [Veklury], favipiravir) were included for mechanistic context. Key host immune components—such as IL-6, IL-1β, TNF-α, IL-10, GM-CSF, CLEC3B, MST1, ITIH2, PABPC1, HSPA7, RBM3, RHOC, MPP1, immunoglobulins, complement component 3, EEF1A1, NRP1, ANPEP, MB, SAA2, SAA1, ITIH3, ITIH4, LBP, LGALS3BP, CFB, CRP, APOA1, ACE2, TMPRSS2, TRIM28, and IL-18—were also integrated

To explore the functional relationships among significant proteins and therapeutic agents associated with HIV/SARS-CoV-2 sample, we constructed a comprehensive protein–protein interaction (PPI) network using the STITCH database, supplemented with manually curated input from known antiviral agents and host immune mediators (Fig. 6). The resulting network revealed a highly interconnected core centered around key cytokines—including IL-6, IL-1β, IL-10, TNF-α, IL-18, and GM-CSF—which function as pivotal hubs driving the host inflammatory response and immunopathogenesis in both viral infections. Notably, other immune-related molecules such as CLEC3B, ACE2, and TMPRSS2 were mapped but remained disconnected from the main network, suggesting potential roles as independent or context-specific effectors in host–virus interactions.

FDA-approved antiviral drugs were included in the network for functional context, including antiretrovirals (tenofovir, lamivudine, dolutegravir, zidovudine) and SARS-CoV-2 treatments (nirmatrelvir–ritonavir [Paxlovid], molnupiravir, favipiravir, remdesivir). These drug entities were placed in proximity to relevant protein interactors, underscoring their mechanistic convergence with host antiviral pathways.

Among the significantly altered proteins, BCAS2 and MELK localized within a cluster of transcriptional and RNA splicing regulators (CDC5L**–**PRPF19 complex), implicating them in virus-mediated dysregulation of host gene expression and cell cycle processes. In contrast, peripheral proteins including PRR11, TEX14, METTL9, NMD3, TUBGCP6, CRISP2, PTX1, and GTPBP8 exhibited no detectable connectivity within the STITCH network, but may nonetheless represent under-characterized regulators or context-dependent effectors potentially relevant to viral pathogenesis.

## Discussion

Proteomic investigations examining the molecular interplay between HIV infection and prior SARS-CoV-2 exposure remain scarce, particularly in the context of chronic immune dysregulation and post-acute sequelae. To minimize the impact of inter-individual biological variability and to generate a representative molecular profile for each cohort, individual plasma samples were pooled prior to LC-MS/MS analysis. This validated approach, common in ‘omics’ studies, allows for the detection of significant differences by reducing the noise from individual variations while also serving as a resource-efficient strategy for high-throughput experiments [12–15].

Ten proteins emerged as key discriminators between HIV-infected individuals with prior SARS-CoV-2 infection and the comparison groups, reflecting diverse molecular processes potentially contributing to pathogenesis. PRR11, a cell cycle regulator implicated in tumorigenesis, may indicate heightened immune cell cycling and proliferative stress during viral immune activation [16, 17]. TEX14 and PXT1, though predominantly testis-specific and lacking established immune functions, may represent germline-derived proteomic spillover or noncanonical functions triggered by systemic stress [18–20]. METTL9, a mitochondrial methyltransferase, is associated with mitochondrial homeostasis and metabolic reprogramming under cellular stress, processes relevant to chronic viral infection [21, 22]. NMD3, a 60 S ribosomal export factor, can be co-opted by viruses to enhance translation efficiency [23, 24]. CRISP2, although sperm-specific, regulates ion channels and may influence systemic ion homeostasis under infection-induced stress [25]. MELK, a serine/threonine kinase, has roles in cell proliferation and stress responses and is linked to immune modulation during infection [26]. BCAS2, a spliceosome component, exemplifies host RNA splicing machinery that can be hijacked by HIV for replication [27]. TUBGCP6 contributes to microtubule nucleation and centrosome integrity—structures frequently remodeled during viral infection [28]. Finally, GTPBP8, a mitochondrial GTPase required for ribosome assembly, suggests that mitochondrial protein synthesis may be altered in prolonged infection [29].

Although TEX14, CRISP2, and PXT1 are classically testis-specific, several mechanisms may explain their detection in plasma. These proteins may appear due to non-specific tissue leakage or germline barrier perturbation during systemic inflammation, a phenomenon reported in other viral or inflammatory conditions [30–32]. Stress-induced ectopic expression or conserved peptide domains shared with somatic paralogs may also contribute to their detection. Additionally, low-level proteomic artifacts, such as background peptide carryover or misassignment within homologous protein families, cannot be excluded. These considerations indicate that their biological relevance remains uncertain and warrants cautious interpretation pending targeted validation.

Collectively, these findings highlight the convergence of cell cycle regulation, mitochondrial function, RNA metabolism, and cytoskeletal remodeling in HIV/post-COVID immunopathogenesis. In a recent study, Zhang et al. [33] reported a proteomic profile in PLWH co-infected with SARS-CoV-2 that differed from our findings, characterized by upregulation of PABPC1, PRIM1, VTN, HABP2, and S100A9, and downregulation of ALCAM, HAS1, CTBS, ANXA3, and ANXA4. These proteins were associated with inflammatory pathways, immune cell migration, degranulation, and complement/coagulation activation.

Several aspects of our dataset align with emerging data describing chronic immune and neurological perturbations following SARS-CoV-2 infection. Acharya et al. [34] demonstrated that even abortive SARS-CoV-2 CNS infection can modulate expression of viral entry receptors (ACE2, TMPRSS2, NRP1, TRIM28) and pro-inflammatory cytokines (IL-6, IL-1β, TNF-α), linking these changes to neurodegenerative pathways such as Parkinson’s disease, Alzheimer’s disease, and amyotrophic lateral sclerosis. The neurodegenerative signatures enriched in our dataset—particularly involving PRR11, METTL9, and MELK—may therefore reflect broad dysregulation of nuclear and mitochondrial bioenergetics and transcriptional stress responses.

Further supporting this interpretation, the upregulation of PRR11 and METTL9 suggests a shift toward a pro-proliferative, metabolically active phenotype characteristic of sustained antiviral responses or unresolved inflammation. Additional proteins that lacked significant STITCH network connectivity (e.g., TUBGCP6, GTPBP8, CRISP2) may represent undercharacterized regulators of stress granule dynamics, innate immune sensing, or post-translational modifications—processes increasingly implicated in viral co-infection biology. These observations remain exploratory and highlight the need for functional validation.

Notably, previous proteomic studies in COVID-19 survivors have demonstrated persistent elevations in markers of epithelial injury, coagulation dysregulation, and mitochondrial stress months after recovery [35]. These patterns correspond with the upregulated cytoskeletal and nuclear proteins in our study, supporting the concept that SARS-CoV-2 can induce long-lasting alterations in cellular architecture and transcriptional control, especially in PLWH.

From a mechanistic standpoint, the nuclear localization of mitotic regulators such as PRR11 and MELK suggests potential involvement in aberrant cell cycle progression or DNA damage response pathways—processes increasingly recognized as mediators of chronic inflammation and tissue dysfunction in both HIV and COVID-19. The inclusion of METTL9, a mitochondrial-associated methyltransferase, further supports the hypothesis that altered bioenergetics and redox imbalance contribute to persistent fatigue and cognitive dysfunction observed in post-acute sequelae of SARS-CoV-2 infection (PASC).

Overall, our findings emphasize the multi-organ impact of HIV with antecedent SARS-CoV-2 infection, where localized CNS disturbances are accompanied by systemic proteomic remodeling. The interplay of virologic stress, immune exhaustion, and metabolic reprogramming likely exacerbates vulnerability to neurocognitive decline. The co-detection of central (IL-6, TNF-α) and peripheral (TEX14, METTL9) markers also suggests that combinatorial biomarker panels may be necessary to monitor disease progression and predict outcomes in co-infected populations.

Future work should focus on longitudinal tracking of these proteins and their correlation with clinical endpoints such as HIV-associated neurocognitive disorder (HAND) severity, PASC duration, and therapeutic response. Mechanistic validation in humanized models or cerebral organoids is warranted to elucidate the functional roles of the novel proteins identified. Given the global burden of HIV and the persistent threat of COVID-19, biomarker-guided strategies may aid in clinical surveillance and the development of host-targeted therapies for this vulnerable cohort.

Although individual-level clinical parameters (COVID-19 severity, duration, PASC symptoms, neurocognitive status) were unavailable, the proteomic patterns identified align with pathways implicated in persistent immune activation, long COVID, neuroinflammation, and HAND. These molecular signatures may represent early indicators of subclinical tissue injury in co-infected individuals.

All participants with prior SARS-CoV-2 infection in this study had fully recovered mild-to-moderate illness. Because severe COVID-19 is associated with markedly stronger inflammatory, endothelial, and mitochondrial disturbances [36, 37], our cohort may be biased toward milder post-infection phenotypes. Long-term immune alterations after COVID-19 have been shown to correlate with acute disease severity [38], suggesting that our findings may not generalize to severe cases in PLWH.

Beyond the proteomic alterations observed, several broader aspects of COVID-19 pathophysiology merit consideration when interpreting our findings. First, the continued global rollout of COVID-19 vaccines and the emergence of novel platforms—including circular RNA–based vaccines—highlight the dynamic immunological landscape in which PLWH experience SARS-CoV-2 exposure [39]. The evolution of SARS-CoV-2 lineages across continents, particularly shifts in spike and non-spike protein architecture, has been shown to modulate viral tropism and host immune activation [40], which may influence downstream plasma protein signatures even after clinical recovery. Diagnostic challenges also remain relevant; delays or inaccuracies in identifying atypical or pauci-symptomatic COVID-19 cases can complicate interpretation of prior disease burden, especially in immunocompromised populations [41]. Additionally, persistent inflammation, autoimmunity, and multiorgan involvement described in long COVID—including endothelial injury, mitochondrial dysfunction, and dysregulated cytokine activity—parallel many of the biological pathways enriched in our proteomic analysis [42, 43]. Finally, advances in pulmonary assessment such as hyperpolarized ventilation imaging [44, 45] underscore the importance of integrating molecular biomarkers with functional diagnostic tools to improve evaluation of post-COVID sequelae. Although our pilot study did not include clinical severity or pulmonary-function measures, these developments provide important context for interpreting the broader implications of the proteomic disturbances identified here.

Despite these insights, several limitations warrant consideration. First, the use of pooled plasma samples, while advantageous for reducing technical noise and enabling deep proteomic profiling, inherently prevents assessment of inter-individual variability and precludes formal statistical inference at the participant level. Pooling may mask outlier-driven biological signals, and heterogeneity—particularly relevant in HIV and post–SARS-CoV-2 immune responses—cannot be evaluated. Although validation of key discriminatory proteins in individual plasma samples would strengthen the findings, this was not feasible because the retrospective specimens were available only in small aliquots insufficient for both comprehensive proteomic analysis and confirmatory assays.

Second, the pooled proteomic profile lacked information on the severity of acute COVID-19 illness among participants. Variability in disease severity could have influenced the circulating proteome and may limit the granularity and generalizability of our observations.

Third, demographic differences—including age and sex—varied across groups and may have contributed to the observed proteomic differences. Age is a major determinant of immune regulation and circulating protein composition, driven by processes such as chronic low-grade inflammation (“inflammaging”) and age-associated mitochondrial dysfunction [46, 47], and large-scale proteomic studies have shown that hundreds to thousands of plasma proteins fluctuate across the lifespan [48]. These age-related immunological shifts could partly account for the elevated expression of cell-cycle, mitochondrial, and stress-response proteins identified in the comparatively older HIV/SARS-CoV-2 group. Sex-based immunological differences may also have influenced our findings. Females generally mount stronger innate and adaptive immune responses and exhibit distinct plasma proteomic baselines due to hormonal regulation and X-linked immune gene expression [49, 50]. Moreover, sex differences have been shown to shape COVID-19 immune responses and disease outcomes [51]. The overrepresentation of females in the HIV/SARS-CoV-2 group may therefore contribute to differences in cytokine-related and transcription-associated proteins. Although ART duration did not differ significantly between the HIV/SARS-CoV-2 and HIV monoinfection groups, long-term ART exposure is known to influence immune activation, mitochondrial homeostasis, and metabolic pathways, even in individuals with sustained viral suppression [52–55]. Therefore, ART-related biological effects cannot be entirely ruled out as contributors to the proteomic signatures observed. Because statistical adjustment was not feasible in pooled analyses, the combined confounding effects of age-, sex-, and ART-related immune variation cannot be fully excluded. The use of pooled plasma samples precluded access to individual-level protein intensities and prevented correlation with participant-specific clinical markers (e.g., CD4⁺ T-cell counts, ART duration, treatment regimen). Consequently, future studies employing individual-sample proteomics will be required to disentangle demographic and treatment-related effects from disease-specific proteomic signatures.

## Conclusion

This study identifies a distinct proteomic signature in HIV-infected individuals with prior SARS-CoV-2 exposure, implicating dysregulation of cell cycle, mitochondrial function, RNA metabolism, and cytoskeletal organization. The findings suggest synergistic host pathway disruption that may drive chronic inflammation and neurocognitive decline. Validation in larger, clinically stratified cohorts is warranted to confirm these candidate biomarkers and guide targeted interventions.

## Supplementary Information

Below is the link to the electronic supplementary material.

Supplementary Material 1

Supplementary Material 2

Supplementary Material 3: **File S1.** Clinical and demographic characteristics of participants, stratified into HIV/SARS-CoV-2, HIV monoinfected, and healthy controls. Data include age, sex, HIV viral load, CD4 counts, comorbidities, antiretroviral regimen, duration of HIV infection/therapy, and relevant SARS-CoV-2 testing dates. **File S2.** Raw proteomic data and significant proteins. Quantitative proteomic dataset including all 13,675 identified proteins, a subset of 100 significant proteins differentiating study groups, and functional classification (PANTHER analysis). Data include UniProt accession, protein names, gene symbols, Gene Ontology annotations, peptide sequences, scores, and intensity values across control, HIV, and HIV/SARS-CoV-2 groups. **File S3.** The Variable Importance in Projection (VIP) box plot analysis demonstrates the protein types that exhibit the highest correlation with the tested sample group, as determined by the sPLS-DA statistics

## Data Availability

The raw MS/MS spectral data have been deposited in the ProteomeXchange repository under the registration numbers JPST004046 and PXD067809 (https://repository.jpostdb.org/preview/57459627568af83c83697a; Access key: 6382). All other data supporting the findings of this study are available within the article and its Supplemental Information files.

## Abbreviations

ACE2: Angiotensin-converting enzyme 2
AMBIC: Ammonium bicarbonate
ARDS: Acute respiratory distress syndrome
ART: Antiretroviral therapy
CD4+: CD4-T-lymphocyte
CD8+: CD8-T-lymphocyte
CNS: Central nervous system
COVID-19: Coronavirus Disease 2019
CRS: Cytokine release syndrome
FDR: False discovery rate
GM-CSF: Granulocyte-macrophage colony-stimulating factor
GO: Gene Ontology
HAND: HIV-associated neurocognitive disorder
HIV: Human Immunodeficiency Virus
LC–MS/MS: Liquid chromatography–tandem mass spectrometry
IL: Interleukin
LFQ: Label-free quantification
NRP1: Neuropilin-1
PLWH: Individuals living with HIV
PPI: Protein–protein interaction
RNA: Ribonucleic acid
RR: Relative risk
SARS-CoV-2: Severe acute respiratory syndrome coronavirus 2
sPLS-DA: Sparse Partial Least Squares Discriminant Analysis
TMPRSS2: Transmembrane serine protease 2
TNF: Tumor necrosis factor
TRIM28: Tripartite Motif Containing 28
VIP: Variable Importance in Projection
