# Supplementary material for: Plasma proteomic signatures in HIV-infected individuals post-SARS-CoV-2 infection
Source: BMC Infect Dis. 2025 Dec 9;26:59. doi: 10.1186/s12879-025-12307-1 (PMC12802228; doi:10.1186/s12879-025-12307-1)

The Variable Importance in Projection (VIP) BOX plot analysis demonstrates the protein types that exhibit the highest correlation with the tested sample group, as determined by the sPLS-DA statistics.


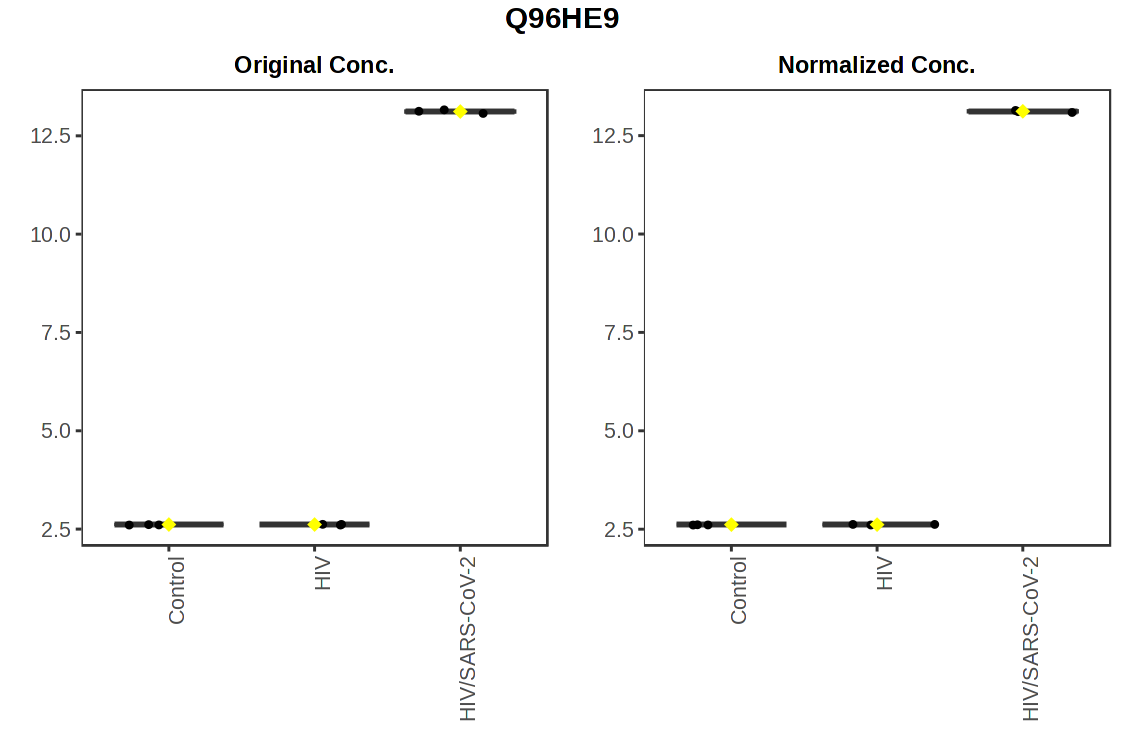

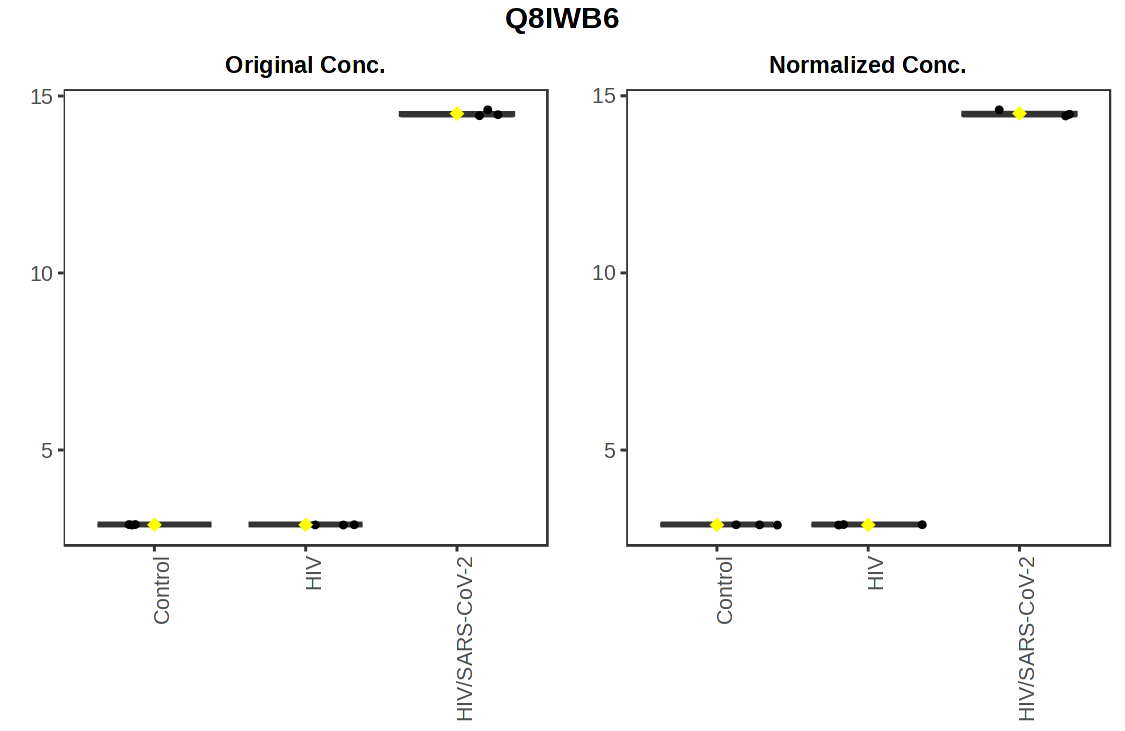


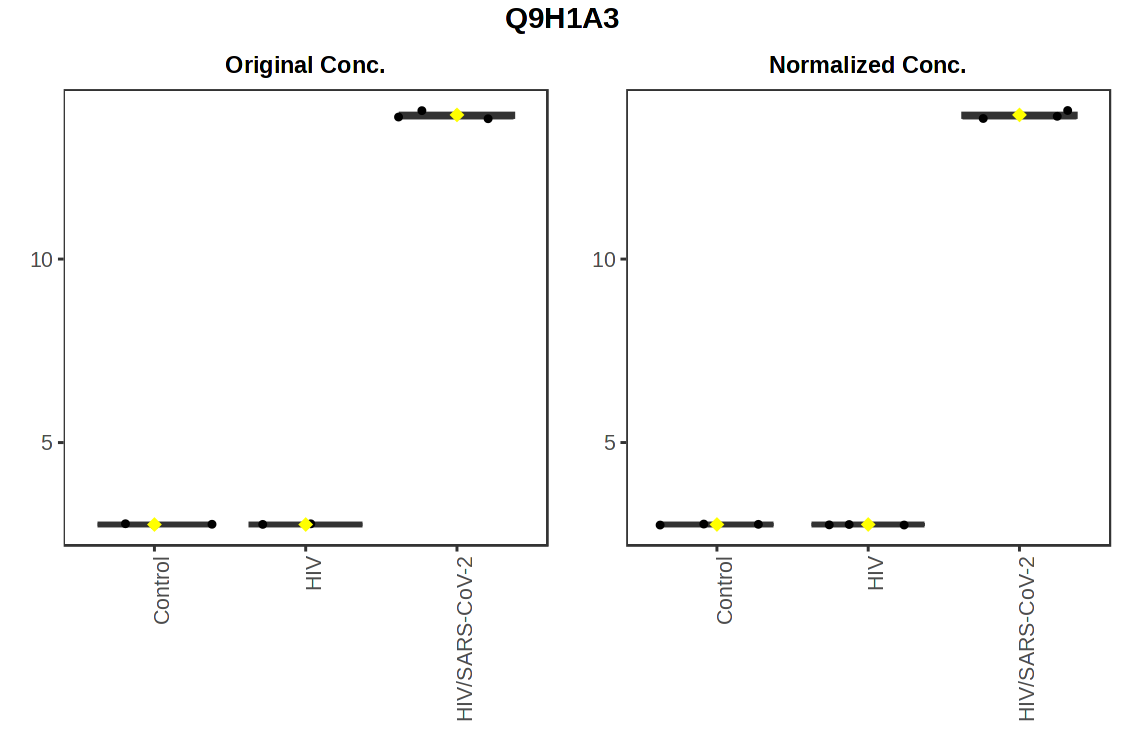

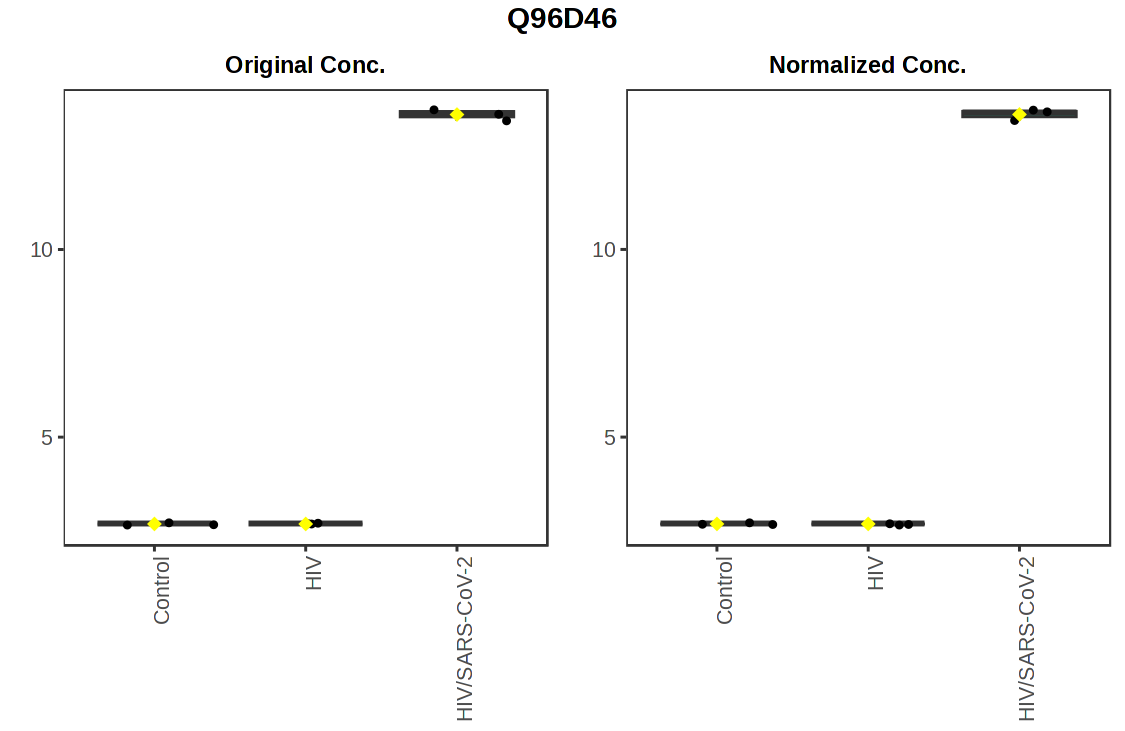

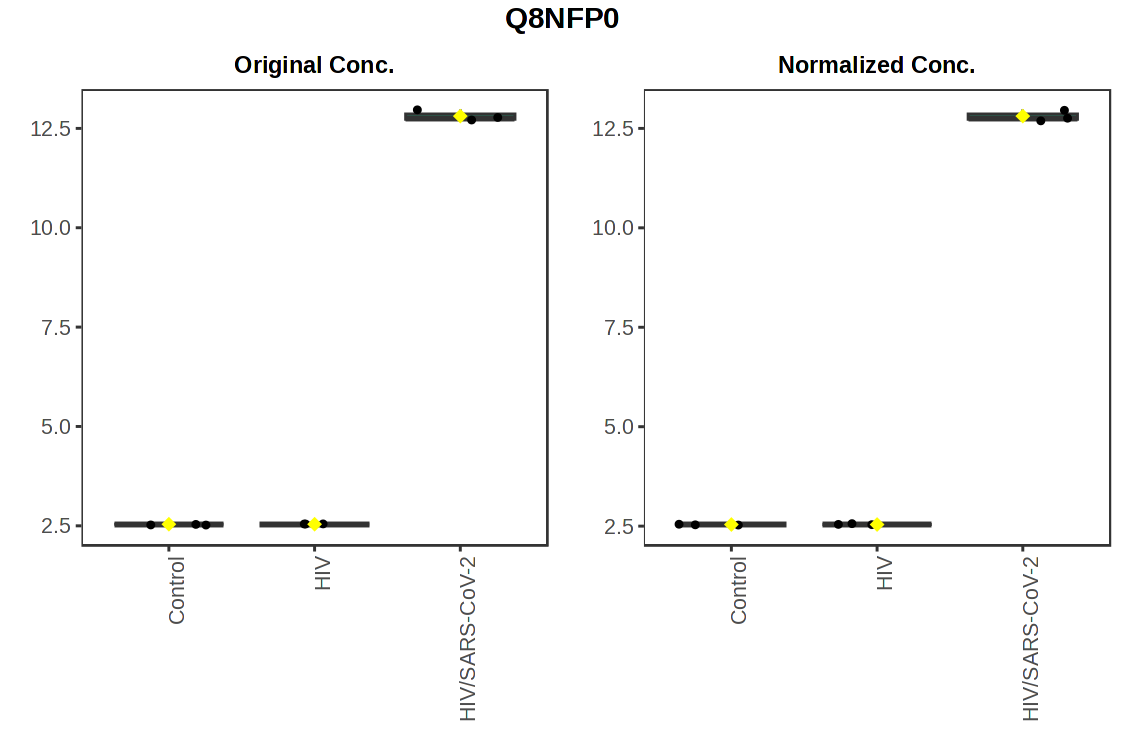

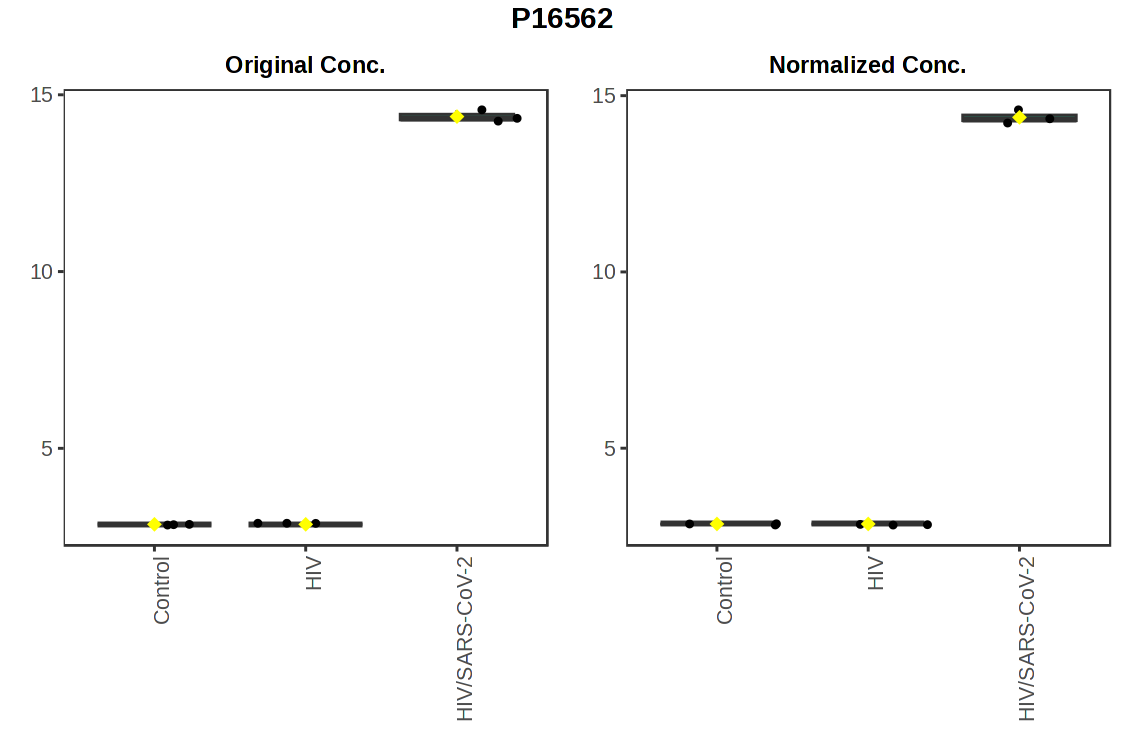

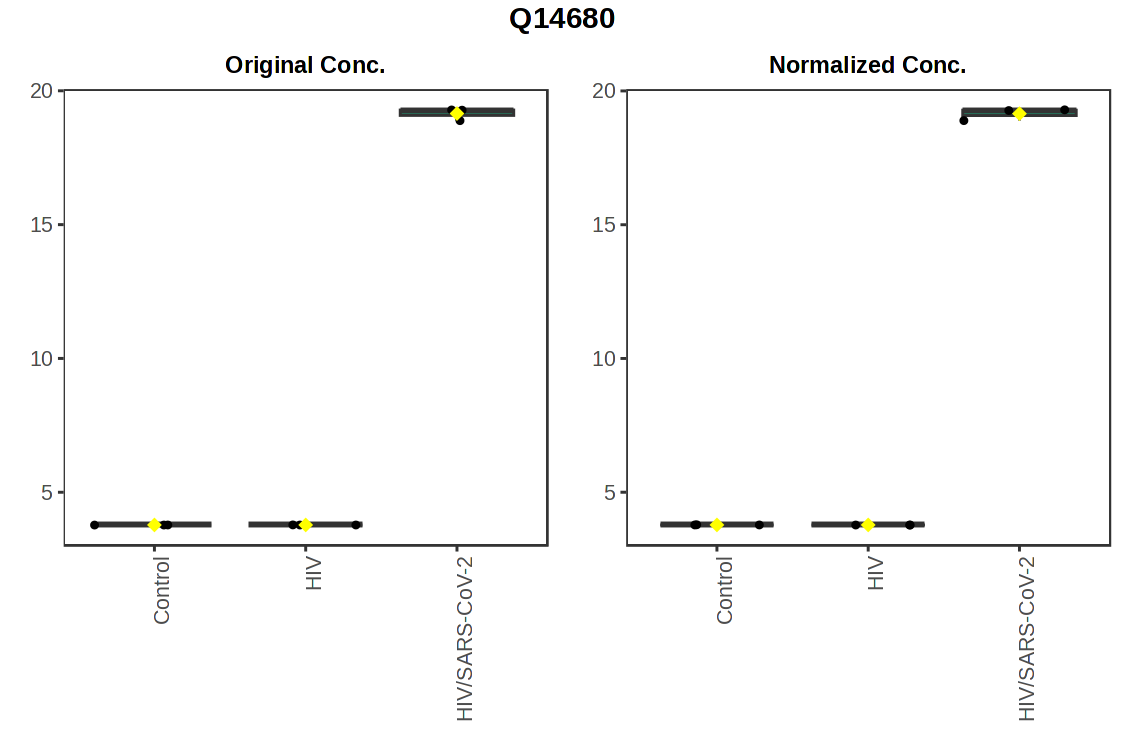

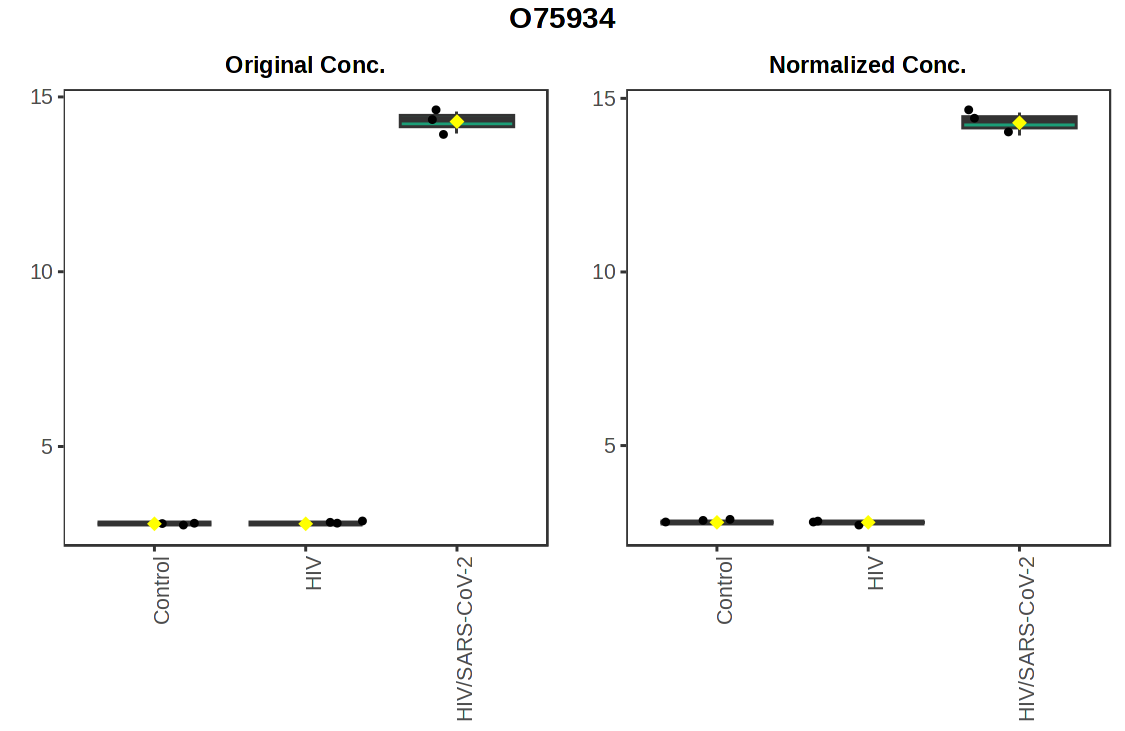

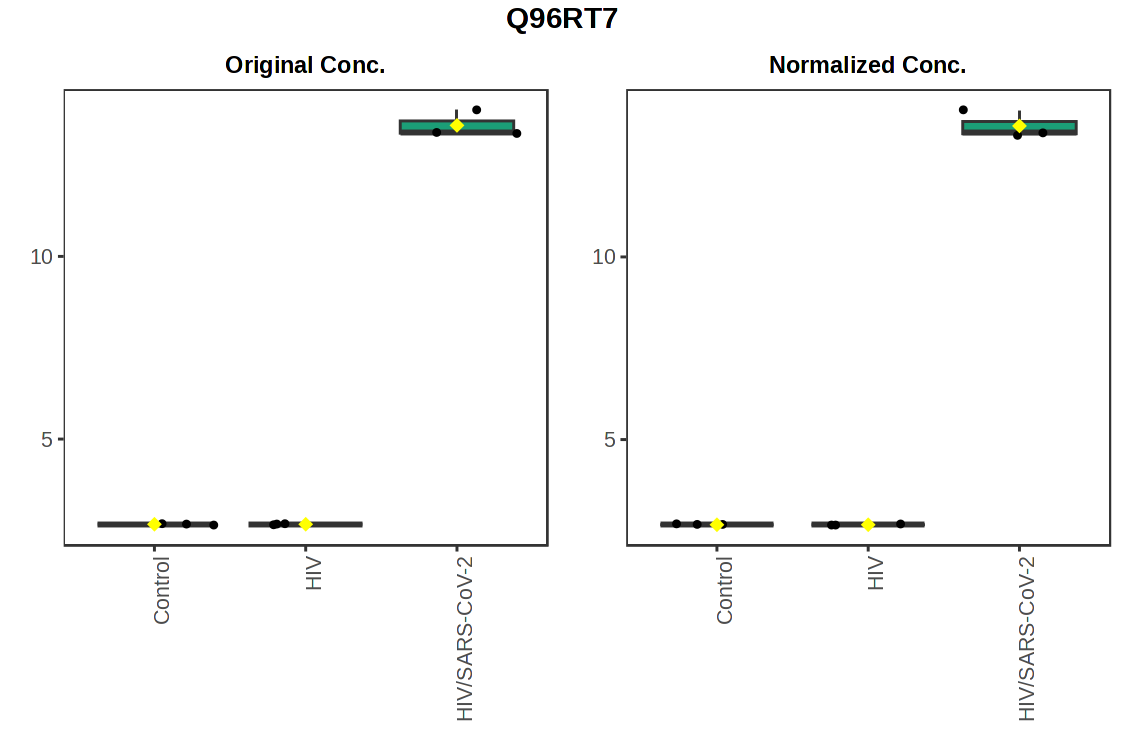

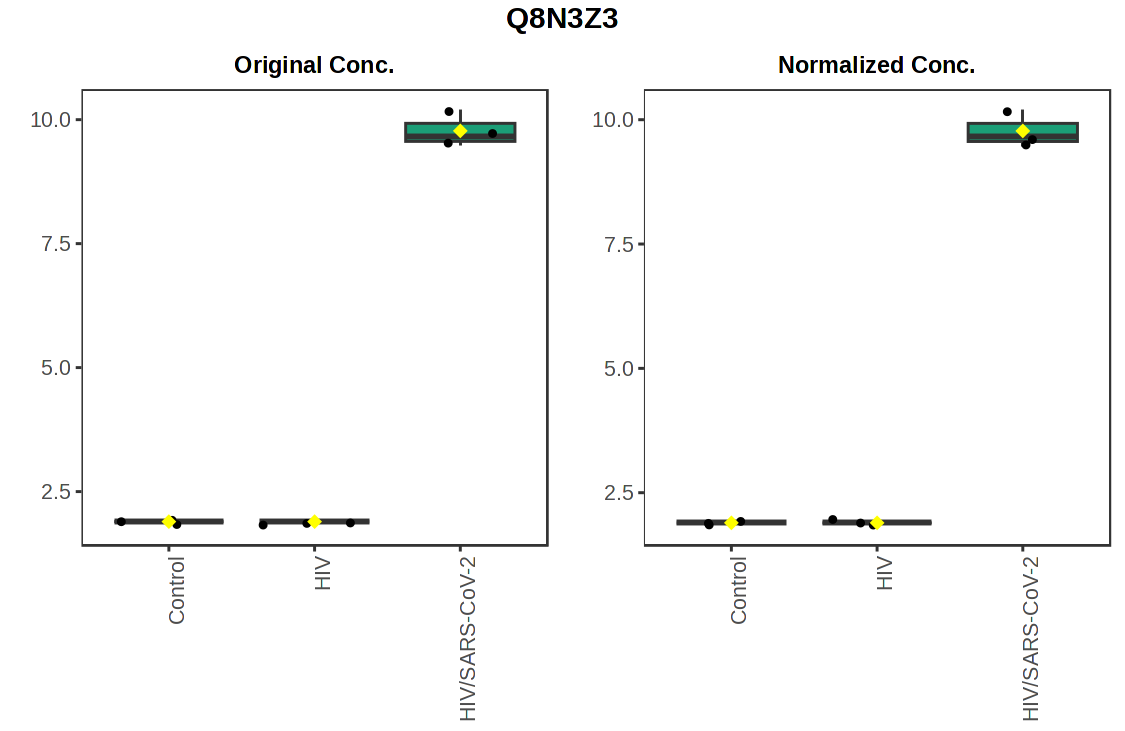

Supplement: Supplementary file 3 — Supplementary Material 3: File S1. Clinical and demographic characteristics of participants, stratified into HIV/SARS-CoV-2, HIV monoinfected, and healthy controls. Data include age, sex, HIV viral load, CD4 counts, comorbidities, antiretroviral regimen, duration of HIV infection/therapy, and relevant SARS-CoV-2 testing dates. File S2. Raw proteomic data and significant proteins. Quantitative proteomic dataset including all 13,675 identified proteins, a subset of 100 significant proteins differentiating study groups, and functional classification (PANTHER analysis). Data include UniProt accession, protein names, gene symbols, Gene Ontology annotations, peptide sequences, scores, and intensity values across control, HIV, and HIV/SARS-CoV-2 groups. File S3. The Variable Importance in Projection (VIP) box plot analysis demonstrates the protein types that exhibit the highest correlation with the tested sample group, as determined by the sPLS-DA statistics [file 12879_2025_12307_MOESM3_ESM.docx]
